# The Effect of Gallium Nitrate on Arresting Blood Flow from a Wound

**DOI:** 10.1155/2011/819710

**Published:** 2011-05-17

**Authors:** Paul H. Goodley, Moshe Rogosnitzky

**Affiliations:** ^1^Department of Orthopaedics, Goodley Pain Clinic, Telz Stone 90840, Israel; ^2^Department of Medical Informatics, MedInsight Research Institute, Telz Stone 90840, Israel

## Abstract

A novel application of gallium nitrate, hitherto unreported, in reducing bleeding time from an open wound is presented. Experiments performed using simple punctures in the forearm demonstrated a very substantial reduction in bleeding time when a solution of gallium nitrate was applied relative to a control. This outcome was shown to be unaffected by the anticoagulant properties of warfarin. The mechanism for such action of gallium nitrate is unknown and merits further investigation, as do the possibilities for such an application to improve both civilian and defense trauma treatment modalities.

## 1. Introduction

Gallium nitrate, the gallium salt of nitric acid, is a drug predominantly used to treat symptomatic hypercalcemia secondary to cancer [1]. The postulated mechanism of action is that it prevents the breakdown of bone through the inhibition of osteoclast activity, thus lowering the amount of free calcium in the blood [2]. It has been shown to be more effective than several other antihypercalcemic drugs [3, 4]. Gallium nitrate has also been used in the treatment of non-Hodgkin's lymphoma [5, 6], multiple myeloma [7], and Paget's disease of bone [8]. To the best of our knowledge, no effect of gallium on coagulation parameters has ever been reported to date.

Here we present a novel action of gallium nitrate, that of stemming blood flow from an open wound via a mechanism of action as yet undetermined. After the serendipitous discovery of the blood-flow arresting properties of gallium nitrate by one of our authors, the authors conducted an experiment on themselves to explore this discovery further and so determine the benefit of gallium in stopping the bleeding from wounds.

## 2. Case Presentation

This study was conducted on two subjects: one (Subject 1, S1) under the influence of warfarin and one not under the influence of warfarin (Subject 2, S2). Subject 1 was on long-term warfarin treatment at the time of the experiment; this was not expected to have a significant effect on primary hemostasis.

In both cases, a puncture of the medial mid-forearm with a number 11 scalpel blade to a depth of 4 mm was made (P1), producing prompt flow of blood as a steady slow stream down the forearm. This was gently wiped with gauze every 30 seconds without applying pressure. The time for bleeding to stop was noted.

A second puncture (P2) was then made following the same method as for the first puncture. At 5 seconds after puncture, once blood flow had commenced, aqueous gallium nitrate (14%) solution (Eby Pharma LLC, Dripping Springs, TX 78620) was applied using a soaked cotton ball. The time for bleeding to stop was again noted.

## 3. Results

The times after which blood flow ceased for each subject and each puncture are noted in Table 1, along with pertinent observations of the presence or absence of clotting.

## 4. Discussion

In this experiment, gallium nitrate substantially reduced bleeding time from an open wound in both a warfarin-treated subject (S1) and nonwarfarin-treated subject (S2) when compared to the control (a reduction in bleeding time from 122 s to 35 s for S1 versus 238 s to 45 s for S2). In this experiment, warfarin did not negate or interfere with the blood-stemming property of gallium nitrate. Interestingly, whilst gallium nitrate stemmed the flow of blood in both subjects, the warfarin-treated subject did not form a visible clot, whilst the nonwarfarin-treated subject formed a very visible brown clot. 

The effect of gallium nitrate described herein requires further investigation and verification as part of a larger, randomized control study, particularly with regards to its mechanism of action (in particular, future experiments should use a standardized bleeding time method such as the Ivy Method or the Simplate II). 

Gallium nitrate has known toxicities when used at higher dosages intravenously, especially over the longer term. Infusion at a rate of around 300 mg/m^3^/d causes renal toxicity and/or diarrhoea in around 15% of patients studied [2]. Microcytic anemia is a possibility, but no suppression of platelets or WBCs is seen; visual or auditory toxicities occur very rarely. However, this present application of gallium nitrate is a single, topical treatment with limited absorption. While questions of toxicity should be addressed in further trials, side effects comparable with intravenous use of gallium nitrate are not expected.

The discovery described in this paper would be of particular relevance to the fields of emergency and defense trauma treatment. One of the current most effective treatments for wounds in these situations is the application of a kaolin-treated gauze (e.g., in the commercial product QuikClot Combat Gauze [9, 10]), which is known to induce coagulation [11, 12] by activation of Factor XII [13]. It may be that gallium nitrate interacts with coagulation factors in a manner similar to current commercial products; it would be important to examine whether this effect takes place only on open wounds or whether gallium nitrate also has an internal procoagulant effect, which would necessitate consideration when it is used as a therapeutic medicine.

This behavior of gallium nitrate would be a valuable addition to such kaolin-based products, as while their usage leads to high survival rates, they do not necessarily stop blood flow rapidly [10]. The possibilities for the use of gallium nitrate in such situations merit further exploration.

## 5. Conclusions

This paper has demonstrated the hitherto unknown ability of gallium nitrate to reduce bleeding time from an open wound. The experiments performed in this case study indicate that gallium nitrate can substantially reduce the blood flow time for simple punctures relative to a control. The mechanism by which this occurs is unknown, although this effect was not interfered with by the anticoagulant properties of warfarin.

This effect should be further explored, both to determine its mechanism of action and to exploit this property for improved management of trauma in both civilian emergency and defense medical situations.

##  Conflict of Interests

One of the authors (M. Rogosnitzky) has a pending patent application on the described use of gallium nitrate.

## Figures and Tables

**Table 1:** The effect of gallium nitrate in reducing bleeding times from simple punctures (P1 and P2) in both warfarin- and nonwarfarin-treated subjects (S1 and S2), with observations.

Subject	Time for bleeding to stop (s)		Reduction in bleeding time (%)	Observations of P2
	P1 (control)	P2 (gallium nitrate)		
S1 (warfarin)	122	35	71%	Small pool of blood stayed about the incision site.
S2 (nonwarfarin)	238	45	81%	Visible clot started to form once flow had ceased.
